# The Present State of Ophthalmic Surgery in France and England Compared Inflammatory Affections of the Cornea

**Published:** 1840

**Authors:** 


### The Present State of Ophthalmic Surgery in France and England compared.

A series of Lectures lately published in a contemporary Journal, as delivered by M. Velpeau, on the Diseases of the Eye, suggest a comparison between the doctrines and practice of the French and English surgeons in reference to ophthalmic surgery. The Lecturer himself commences by defending his own country from a presupposed charge of inferiority to Germany and England, and throughout the lectures, there is instituted a running comparison with the two countries, and not always, we think, a just one. From the tenor of these discourses therefore, we were insensibly led to the analysis of differences in theory and practice between the French and English hospitals and the refutation of some doctrines, which it seemed to us were founded in error. From this half-unconscious labour we soon became convinced that, by a more systematic analysis of the contents of the whole series of lectures, useful facts might be elicited, and interesting conclusions drawn, for the benefit of our readers.

Leaving the question of who first led the way to the classification and more accurate study of the diseases of the eye —as one of only remote interest— we proceed at once to the surgeons of the present day, who have in both countries devoted some portion of their time, more especially to these diseases, and who, by their writings, have contributed to the advancement of this fragment of surgical science.

In reference to English surgeons M. Velpeau remarks, " we cannot, I confess, bring forward so many pure ophthalmologists, but I appeal to your reason, is it necessary to be decorated with the name of oculist to have exact notions respecting diseases of the eye? Do not these affections belong to pathology in general? And ought not every good surgeon to be acquainted with this branch of his profession as well as with all others?"

That it is not necessary to be decorated with the name of oculist in order to have exact notions respecting diseases of the eye, we not only most cordially agree, but without a knowledge of pathology generally, we hold very lightly any " notions" the pure oculist may entertain. It is precisely, therefore, for those English surgeons whom M. Velpeau quotes, and many to whom he does not allude, that we claim a high merit, if not a ground of superiority over their neighbours. They were among the first to rescue from the hands of mere oculists this important class of diseases, and replace them within the limits of general surgery. Thus the Lecturer would scarcely term " pure ophthalmologists" Travers, Mackenzie, Wardrop whom he quotes, or Lawrence, Guthrie, Tyrrel, Middlemore whom he does not mention. They have made the most valuable contributions, and indeed effectually proved that general scientific attainments, and the knowledge and practice of surgery in its most comprehensive sense form the best guarantees for the sound and judicious practice of this particular branch.

However, an invidious comparison between the individuals of different nations is by no means our object—but by analysing the doctrines of the most eminent surgeons of each, to trace out differences in practice—balance their respective merits, and so arrive at useful conclusions.

We are told " the school which reigns in France at the present day is that of Beer—it reposes chiefly on two principles—1. Diseases ought to be classed according to the natural system—that is, according to their physical symptoms—2. The classification should also be founded on the nature of the disease which may be considered the consequence of the first. "Such are the principles which have given birth to *scrophulous, rheumatic, and catarrhal ophthalmia.*"

From these principles it appears that M. Velpeau entirely dissents—that is, "he differs entirely from those who maintain that a disease of the eye assumes peculiar symptoms because the patient's constitution is modified by a general affection."

Although a little farther on we find that he acknowledges the influence of general affections upon the eye, and believes that they exercise the same influence over these as of all other diseases, yet he conceives the division of ophthalmia into scrophulous, rheumatic, and catarrhal, is altogether erroneous. The whole of this strong difference of opinion, and the whole error he attributes to Beer and his followers, he declares to be simply this, "they judge of the constitution by the appearance of the eye, whilst we ought to judge of the eye by the constitution."

This strikes us very strongly as little better than a jingling of words, wherein there are great distinctions with small differences.

By what means do we ever judge a patient to be scrophulous—is it not by the development of the disease in some tangible form? If a child is brought to a surgeon with a thickened lip—tumid belly—enlarged glands in the neck—or a slow enlargement of the knee with little pain, without any obvious cause, he forms his opinion that these are the signs of a scrophulous habit, and directs his treatment accordingly. If many of these signs are wanting, but instead there is an affection of the eye marked by excessive intolerance of light—a disease of long standing—with considerable action, but little power—on which local treatment produces no effective amelioration—is it not equally rational to assume this to be a type of scrofula developed in that organ?—and if our treatment, based upon this assumption, leads to the rapid amelioration and ultimate cure, are we not warranted in believing the diagnosis correct? Where then is the error of judging of the constitution by the character and progress of a disease in the eye. But, says M. Velpeau, "nothing is easier than to give rise in the healthy eye, by artificial means, to an inflammation assuming all the symptoms of the scrofulous, &c."

We confess we are exceedingly sceptical on this point, and certainly, during a tolerably extensive experience in the treatment of ophthalmic patients, we can recall to mind no observation to confirm it. Doubtless every scientific surgeon will draw his conclusions from the general state and appearances of the patient, in addition to any specific disease in the eyes; but should no other indications exist, would M. Velpeau then reject the evidence of these diseased organs and treat them as a mere local affection?—if so, the prognosis would assuredly be of the most unfavourable kind—and but a very short time ago we could have furnished him with a case strictly scrofulous in all its characters, which yielded only to treatment based upon such diagnosis, and having previously resisted all other prophylactic measures, although no other signs of scrofula were manifest.

M. Velpeau would always judge the eye by the constitution, and doubtless so do those from whom he expresses so strong a dissent—if there be a difference between him and his confrères of the French school, it amounts simply to this, they take into their consideration such evidence as the diseased organ presents, in addition to any constitutional signs, while M. Velpeau entirely rejects it—admitting the while that "general affections exercise the same influence over diseases of the eye that they exercise over all other diseases."

To this it seems, so far, are M. Velpeau's claims to originality restricted, and as we cannot altogether believe that a surgeon of his standing and experience really rejects, as he would imply, the index which the principal disease affords to the constitution, we may for the present consider the *difference* in his opinions of no very solid character, and take him as our guide to the doctrines and practice of the French school of the present day.

These lectures are first devoted to the inflammatory diseases of the eye-lids and globe, laying aside every thing relating to degenerations of tissue, and we are farther reminded that he does not take into consideration the constitution of the patient or the different specific causes of disease! Sounds this not remarkably like the announcement of the part of Hamlet—left out by particular desire! If indeed this be the mode in which the diseases of the eye are studied and treated by the French school, then we might assuredly at the outset congratulate our countrymen on a difference of the highest importance, for no Lecturer of the present day, we think, would attempt to instruct his pupils by setting aside all considerations as to the constitution of the patient, or the different specific causes of disease, and indeed M. Velpeau, as early as the second lecture, abandons his own plan.

He groups the different inflammatory diseases of the eye-lids under the general term of blepharitis, presenting varieties according to the nature of the tissue primarily affected, and to the variable intensity of the inflammation—these he further defines into five classes.

The classification of the various forms of blepharitis into *mucous, glandular, granular, ciliary,* and *purulent,* offers little novel or worthy of remark; it is generally adopted in Europe. The granular form of blepharitis is erected into a distinct class or form of inflammation instead of being arranged, as it is almost invariably observed—viz. as a consequence of catarrhal or purulent ophthalmia. The catarrhal disease itself commences in the mucous follicles of the conjunctiva palpebralis, and when it has continued for a certain period, the papular state denominated granular results; but it is maintained in these lectures, that there is a peculiar, "a granular inflammation," which, in common with English surgeons generally, we cannot admit.

Again, with regard to the purulent blepharitis of new born children, M. Velpeau observes that this has been improperly confounded with purulent ophthalmia, with the Egyptian, and gonorrhœal. Their unity is objected to on the grounds that the two latter do not so much attack the palpebræ as the eye itself, and the purulent ophthalmia of infants is almost entirely confined to the conjunctiva palpebralis.

In England it is pretty generally taught that these are essentially the same disease, varying greatly in their degrees of intensity or virulence, but the same in site—the same in leading characters.

Nor can we for a moment admit the accuracy of the view set forth by the

Lecturer; we maintain that the Egyptian and gonorrhœal ophthalmia, as certainly as the purulent of infants, commences in the lids, involves them *first*,—that in infants, if not speedily arrested, it spreads to the globe of the eye, destroys the cornea, and in similar manner to the Egyptian and gonorrhœal, leads to the destruction of the organ. The *catarrhal*, the *purulent ophthalmia of children*, the *Egyptian* and *gonorrhœal*, form but one family of diseases, having the same seat, course, and effects in proportion to their degree of intensity arising often from apparently different causes—yet all more or less contagious, and capable of communicating a purulent disease, the precise characters or virulence of which will be variously modified by the patient's constitution and other general circumstances.

For the treatment of these forms of blepharitis, the therapeutical means are divided into direct and indirect, which by English surgeons are termed constitutional or general, and local.

The opinions expressed as to the total inefficiency of general treatment to arrest the progress, or remove these diseases, closely coincides with the opinions and practice in England; but when we examine the local applications, upon which consequently our neighbbours rely for the cure, although there is a general praise of stimulants and escharotics; in some of the severer forms, their use seems too timid in many instances to do all the service of which they are capable, particularly in the purulent forms.

For *mucous blepharitis*, the solutions of nitrate of silver, sulphate of zinc, and sulphate of copper are preferred, particularly the first; to commence, however, with half a grain of arg. nit. to an ounce of water we should deem a waste of time; two grains to the ounce we believe the lowest curative strength. In the glandular inflammations the ointments are preferred. In what is termed the granular blepharitis, M. Velpeau, after trying the various collyria, and finding them totally inadaquate; in 1831, it seems, began to try that which had been determined twenty years before in England to be the only efficient practice—viz. the solid nitrate of silver and sulphate of copper. Not generally successful in his hands however, these he renounced for a powder containing sulphur and calomel, and finally concludes, that a really efficacious treatment is yet to be found, and recommends a scrambling treatment of bleedings, blisters, collyria, and a weak unguent of nitrate of silver; and when all fail, again the solid nitrate of silver, or sulphate of copper.

The experience of every surgeon whose attention has been given to these diseases will bear us out when we say, that the treatment of granular conjunctiva offers no peculiar difficulties with the general constitutional measures indicated by the state of the patient's system, and the methodical and judicious use of escharotics and counter-irritants; it is a disease which always yields, and an incurable case would be deemed a great reproach with us.

In the purulent ophthalmia of new born children, the principle of treatment is well laid down, and offers no novelty in reference to English practice; but again, the application fails in some degree, and in one particular point we must believe is erroneous, and calculated to do mischief. In the chronic stage methodical *compression* is recommended; we have observed this to be a plan unfortunately adopted without instruction generally by the nurses and mothers, and nothing but injury have we ever seen result. In these cases too the most efficient mode of applying the nitrate of silver is neither in its solid form nor in solution, but as a strong ointment, gr. x. to ʒj. of lard, and this in the melted state, smeared over the conjunctiva palpebralis by a camel hair pencil—this, for the most severe forms, we have invariably seen most beneficial and efficient in its action. This has been both demonstrated and taught in the Westminster Ophthalmic Hospital during the last twelve years and more, and as Mr. Guthrie has been often attacked for the too lavish and indiscriminate use of this remedy, it is but fair that he should have any merit due for its bold application. When the disease is mild a weak solution answers all the purposes.

The same observation applies to the two remaining varieties of purulent ophthalmia, the Egyptian and gonorrhœal, the principal and primary seat of which, M. Velpeau insists is in the sclerotic conjunctiva—but as these, especially the latter, run a more rapid and disastrous career, no surgeon in England would think himself authorised in relying entirely upon the effect of a local remedy, and consequently free depletion, counter-irritants in the vicinity, and medicines which have a direct tendency to lower the circulation and depress the powers of the system generally, are combined. We have never seen a case where this general and local treatment, the nitrate of silver, effectively applied in the manner indicated to the whole surface of the palpebral conjunctiva, has failed in saving the eye, if, when the treatment began the cornea was safe. Without this local treatment, the value of which however is not we believe so generally appreciated as it deserves, we are convinced that, in the majority of violent attacks, no general treatment, however prompt and energetic, will suffice to arrest the disease and save the eye. The treatment as laid down in these lectures we must therefore conclude to be inefficient and inferior to that which is taught and practised by many in England

On conjunctivitis or inflammation of the sclerotic conjunctiva there is little in the French theories or practice worthy of remark. The same principle is very properly enforced there as here, that general measures form the adjuvantia—and local remedies should occupy the first place in the treatment of these forms of ophthalmia. We rejoice to see that in some of the diseases, if not in others, the French students are taught to estimate the nitrate of silver at its true value in the treatment.

We could wish that even in England, where it has some strenuous advocates, its importance as a remedial agent were more generally acknowledged and understood. We speak confidently, having watched its effects in many thousand cases.

In the treatment of chemosis our neighbours would seem to be halting somewhat behind ourselves. M. Velpeau speaks slightingly of scarifications—strongly recommends general depletion and the application of leeches—but it seems is unacquainted with the very effective treatment which of late years has been adopted—first we believe in the Moorfields institution—viz. completely dividing with the edge of a knife the swelled and injected tissue in radii, from the edge of the cornea taking the pupil for the imaginary centre. It is but a short time back that we saw Mr. Tyrrel by this operation most completely and effectually relieve the disease, and remove all danger to the cornea within a very few minutes' space. Scarifications, as generally understood, deserve to be spoken of as lightly as M. Velpeau has done—we must presume therefore that the mode of treatment just described is unknown in the French Hospitals.

### INFLAMMATORY AFFECTIONS OF THE CORNEA.

Intolerance of light and epiphora are dwelt upon as signs distinctive of keratitis, iritis or retinitis, and it is broadly stated and even lengthily argued, that these two symptoms never take place in pure conjunctivitis. This is contrary alike to observation and physiological facts. How is it that, if any irritating particle enters the eye, there is an immediate discharge of tears? The anatomist accounts for it at once—the lachrymal branch of the 1st. division of 5th. after supplying the lachrymal gland, sends filaments to the conjunctiva and orbicularis;—thus providing for that intimate sympathy which exists among these parts. To maintain therefore that an increased discharge of tears does not accompany and follow the acute irritation of conjunctivitis, and therefore is not a symptom, is to theorize against all observation. There is no doubt also that the inflammation of the external tissues frequently occasions a sympathetic increase of sensibility in the internal textures, producing various degrees of intolerance of light. To assume therefore that *photophobia* and *epiphora* are so distinctive of inflamed cornea that they never exist in irritations or inflammations of the conjunctiva solely, is clearly an error.

We need not follow the Lecturer through his definitions of acute and chronic keratitis—the superficial, interstitial, and deep-seated, they are generally good and accurate, in nothing differing however from the doctrines taught in our own schools, and indeed the study of the diseases of the cornea, as a separate tissue, was first most successfully cultivated in England by Mr. Wardrop.

Diagnostic signs are founded upon the colouration of the cornea, which, neither to the same extent nor in the same manner, are recognised in England. We confess that, to our minds, the definite character given to these changes assume somewhat of an imaginative character. Thus—

"*Coloration of the Cornea.*—Returning to the consideration of the anatomical symptoms of keratitis, we must now study the changes which take place in the colouration of the cornea, when it has become the seat of inflammation. When examined in the different stages of the disease, it will be found that the cornea may be of four distinct shades or tints; with these it is important that you should become acquainted, as they will greatly assist you in the diagnosis of the various forms of inflammation we shall have to study.

*The water-green tint*—The shade to which this term is applied is met with during the first period of the inflammation, and is not easily distinguished at first sight. Indeed, to form a correct estimate of the change that has taken place, the cornea must be attentively examined, and that on a patient who has only one eye affected. I describe the appearance which it then presents, under the name of the water-green tint, from the likeness which exists between the colour of the cornea and that of a sheet of water spread over a large surface, the transparency of which it very faithfully represents. Viewed sideways, and partially shaded from the light, the cornea has also a peculiarly moist humid appearance. These phenomena are not, in my opinion, to be attributed to any change in the tissue of the cornea itself, but to a change in the aqueous humour of the anterior chamber. I do not, however, attach much importance to this explanation.

*The brown tint.*—When the inflammation continues, the cornea soon becomes of a light brown colour. On examining it attentively, we also generally find that it is covered, either entirely or partially, with a great number of exceedingly small granulations, smaller even than those which I described as existing in granular conjunctivitis. These granulations give the surface of the cornea a rough and uneven appearance, which we might aptly compare to that of a mucous membrane in the healthy state, and constitute, no doubt, what has been described by M. Lepelletier under the name of granular ophthalmia. The change which takes place in the colour of the membrane evidently depends on an alteration of its tissue—an alteration which may be referred to the external lamellæ. We shall also see presently that it is a symptom of superficial keratitis.

*The yellow tint.*—A yellow colouration of the cornea is a far more serious symptom than any we have yet examined, experience having shown that it always indicates a deep-seated affection of the tissue of the organ. The yellow tint first appears as a speck in the centre of the cornea, or as a narrow band or circle round its circumference. This circle may be complete or incomplete ; in the latter case, it is generally found to occupy the inferior portion of the cornea. It is important that you should become familiar with this symptom, as you might otherwise mistake it for a natural appearance caused by the reception of the cornea within the anterior border of the sclerotica. Such an error would probably be attended with disastrous consequences, the rapidity with which the yellow coloration extends being such, that unless active measures are adopted to arrest its progress, the entire cornea is invaded, and vision is nearly always more or less injured.

*The dark-red tint.*—This epithet does not convey as clear a notion of the colour which it is intended to describe as the expression adopted by Mr. Wardrop. He calls it the flint-stone colour ; and those of you who have paid attention to diseases of the cornea, and have been able to observe it in the state the word represents, must confess that it is strictly appropriate. The change in the colour of the cornea generally commences, as with the yellow tint, in the centre."

These we repeat—as far as our own experience enables us to decide—have been elaborated to the above number and distinctness, with some little aid from the imagination.

With respect to the treatment, the same principles are taught as those acknowledged in England—viz. that here first commences the abandonment of means, chiefly local and adapted to the various diseases of the superficial tissues—for the general treatment required for the inflammation of the internal textures. The cornea is indeed the link, as it were, between the two —in its superficial covering belonging to the first class, in its deeper structure essentially to the second. Hence the peculiarity, that occasionally local, but much more frequently general treatment is chiefly to be relied upon for subduing the inflammation, while in some few cases the two methods are nearly equally required, and upon their judicious combination the cure essentially depends.

Among the therapeutical instructions, however, we find some difference from the English practice not unworthy of remark.

The first consists of the application of blisters over the eye, of which M. Velpeau speaks highly, although he confesses that it cannot be extensively adopted in private practice.

"I have employed blisters over the eye more than a hundred times, in every form of inflammation, principally with a view to ascertain when they ought to be used. I have found them an extremely useful remedy in conjunctivitis, in superficial and in deep-seated keratitis, and especially in ulcerated keratitis. The counter-irritation they produce appears to dissipate the congestion of the tissues; to arrest or prevent the effusion of coagulable lymph, and to modify the ulcerations if any exist. They are not, on the contrary, very beneficial in blepharitis, purulent ophthalmia, in inflammation of the interior of the eye, nor even in keratitis when the inflammation has assumed the chronic form."

The following directions for their use are worthy of attention :—

"When blisters are applied over the eyelids, some precautions are necessary. The patient must close his eyelids gently, without contracting them, as they would otherwise be more or less folded, and the blister would not cover their entire surface. The blisters must be then carefully applied, in such a manner as to be in contact with every portion of the external surface of the palpebræ, and over it is placed some lint or cotton, to fill up the cavity of the orbit, in order that compression may be equal. A bandage carried a few times round the head and over the eye will suffice to keep every thing in position. You will generally find the eyelids much swollen the next day; when the blister is taken away it must be dressed as usual without any attempt being made to see the eye. In the course of two or three days the swelling diminishes—the eyelids become hardened, and the eye may be freely examined. Although the pain generally diminishes in the course of a few hours, the amelioration produced by the blister is not very sensible for a day or two. The symptoms are then found to abate, and they continue to do so for about ten days, when it becomes requisite to repeat the blister. It is necessary that you should be aware that the amelioration does not take place at first, as you might otherwise be led to despair of any effect being produced, and employ some other remedy to which the subsequent improvement would be attributed.

In conclusion, blisters over the eyelids seldom effect a cure alone, but they nearly always diminish the violence of the disease, even when every other plan of treatment has failed to do so, and thus enable you effectually to overcome the malady. I have repeatedly found this to be the case in obstinate inflammatory affections of the cornea accompanied by ulceration."

A more elegant and infinitely less troublesome application would be to paint the lid and orbit with the liquor lyttæ—the degree of counter-irritation could be accurately regulated, and this mode of blistering, quite as effective, would be much less open to the patient's objections.

Again, with reference to calomel and tartarised anatomy, the English practice meets with no advocate in the French Lecturer—with whom we are compelled to join issue. Long continued observation of the utility of these remedies, in cases probably not less numerous than those to which he refers, has led to a conclusion opposed to the opinion here expressed.

"As regards calomel, the panacea of some ophthalmologists for diseases of the eye, I have given it in every possible manner. The result of all these essays is, that I cannot allow calomel to be as efficacious a remedy as English practitioners represent it to be. The efficacy of this remedy has in my opinion been much exaggerated, it is in reality much less beneficial in keratitis, and indeed in ophthalmia in general than is commonly asserted.

"Tartarized antimony has been much praised in England by Mr. Lawrence and Dr. Mackenzie. I have myself often tried it in doses of from four or six to 10 or 15 grains, but do not believe that it exercises any specific action whatever over the disease."

The opinion on the first of these remedies is opposed to that of the majority in this country. On the value of ant. tart. English surgeons are by no means agreed.

With the following paragraph, on the contrary, we cordially assent—and although these principles have long been well understood and practised in our eye-hospitals, they are valuable enough to bear reprinting.

"However successful the general treatment of acute keratitis may be, it seldom entirely subdues the inflammation. Local treatment is not however equally serviceable in every form of keratitis. When the deeper layers of the cornea are inflamed, their influence must necessarily be very limited, as they do not remain in contact with the cornea sufficiently long to reach the seat of the affection either by imbibition or by endosmosis. Some ophthalmologists have been so much influenced by these considerations as entirely to reject local remedies in the treatment of keratitis. This rejection however is not warranted by experience, as even in diffuse interstitial keratitis the application of local remedies is not unfrequently followed by favourable results. In superficial keratitis they are extremely useful, especially when the conjunctiva is inflamed—and this is nearly always the case—by dissipating the inflammation of the conjunctiva. They also act on the affection of the cornea. This you will at once understand if you recollect the nature of the vascular communication which exists between the interior and the exterior of the eye. When ulcerations of the cornea exist, more benefit is to be derived from topical applications than any other class of remedies."

Passing on to the sequelæ and complications of keratitis, several forms of softening of the cornea are dwelt upon, which do not find place in our best works on surgery, nor have they attracted any general attention on this side the Channel. We give, therefore, the words of the Lecturer.

"I have occasionally met with a species of softening of the cornea, which is but imperfectly known; indeed I do not know whether it has ever been properly described. The cornea, the tissue of which has become exceedingly rarefied, forms between the free margins of the eyelids, a black, brown, or reddish tumor, projecting like a large staphyloma of the iris. This tumor is soft, insensible, and radiated. I first observed it in two women, nurses at the Hopital de la Maternité, who were both affected with a chronic vaginal discharge. They were both of a lymphatic constitution, deteriorated by poverty and bad living, and had been attacked, without any appreciable cause, with violent purulent ophthalmia. The flaccidity of the muscles, the copper-coloured appearance of the face, and the vaginal affection, led me to suppose that they were affected with syphilis, although they positively denied this to be the case."

These various forms of softening of the cornea are nearly always attended with serious consequences to the functions of the eye, the cornea being generally more or less deformed—even in the most favourable cases vision is necessarily disordered.

The ulcerations of the cornea, which are divided into five kinds, offer no peculiarity of description worthy of note, if we except the incised ulcer, described by Mr. Lawrence in his work on Venereal Diseases of the Eye, as a symptom of venereal ophthalmia, and which M. Velpeau states he has often met with, when he could not possibly attribute it to a venereal affection. But, on this subject, after his declaratory preface, we are naturally prepared to find him sceptical. A very proper distinction is made with reference to the treatment between ulcers the progress of which depend on a continuance of the corneal inflammation, and those which seem to be a source of irritation and inflammation. The definition of the three species of opacity has the merit of great pithiness.

" The *nebula,* in which the opacity occupies only the superficial lamella of the cornea—*albugo,* in which the middle layers are also affected—and, lastly, *leucoma,* in which the entire thickness of the cornea has become opaque."

With these remarks we pass on to the inflammations of the internal tissues—and first, of the sclerotic. Here we are startled by the assertion, that there is no such disease as sclerotitis—speaking of the pink zone of the sclerotic vessels, he says :—

" The interpretation which I give to this phenomenon, is very different from that which is generally adopted by ophthalmologists. I consider it as indicating the presence of keratitis or iritis, whilst they look upon it as symptomatic either of a specific inflammation or of sclerotitis.—A disease the very existence of which I think improbable."

Is this an attempt on the part of the Lecturer, by a startling contradiction to a very generally admitted fact, to attract notice as an original thinker ? It is difficult to understand how M. Velpeau, with his opportunities for observation, can have passed several years in the study of ophthalmic cases without having met with the most direct contradiction to his opinion, in the eyes of his patients.

He is firmly convinced that the vascular zone and other symptoms described by authors as indicating the presence of sclerotitis, are merely the result of an inflammatory affection of the cornea or the iris. And what if the zone and other signs are most evident, distinct, and undeniable, with the iris perfectly natural in all its appearances—giving no one indication of inflammation—the cornea clear and bright, and equally free from any shade of disease? Can this be termed iritis—or corneitis? or is it not palpably and demonstrably a disease of the sclerotic tunic? Such a case is at this moment under our observation, giving in every particular so strong a refutation of M. Velpeau's opinion, that we confess we were startled on first perusing the observation and began to look for a misprint.

Many such cases we have seen—nor even in France does the existence of sclerotitis seem to be called in question, save by M. Velpeau, who, in searching for the original, seems to have lost sight of the real and the palpable. His analysis of the facts on which he assumes the existence of sclerotitis to be founded by its advocates, scarcely requires refutation. He first, commences by showing that iritis and corneitis will produce the injected zone—that the tunic between the vessels is white—that he has failed in producing inflammation by wounding the sclerotic, although he perfectly succeeded by injuring the cornea.

Doubtless iritis and keratitis will produce the effect—but how account for it when neither of these exist? Secondly, the tunic cannot, to the best of our belief, be seen white between the radiating vessels of the zone, as is asserted; for, in the case above quoted, we have most carefully examined. Thirdly, that he should fail in producing this zonular inflammation by a wound of the sclerotic, which is quite capable of inflaming the cornea and thus producing it—what does it prove, but that which is known to a junior student, viz. that the cornea is much more susceptible than the sclerotic coat. In this most astute departure, therefore, from the German and the French school, founded upon it, we cannot but conclude M. Velpeau's originality to be particularly infelicitous, and based upon error of the most demonstrable character.

Not less are we at issue with the Lecturer—who again represents his individual opinions only—on the non-existence of arthritic, rheumatic, and scrophulous ophthalmia as specific diseases. This is a question of some importance, and as we entertain the conviction that much mischief would result were the profession generally to embrace the opinions disseminated in these lectures, we shall inquire into M. Velpeau's grounds for supporting them.

The Lecturer questions, in the first instance, the existence of gout, of scrofula, and of rheumatism, as specific diseases in the system—and proceeds to say that, having frequently met with these diseases where no such general complaint had ever existed, the obvious inference is, that they are not specific or depending upon a peculiar and specific disease of the system.

M. Velpeau is not the only one who has put forth this opinion in opposition to the doctrines of Beer and the German school generally.

Dr. Mackenzie, in his excellent work on the eye, says he has often been led to doubt whether arthritic ophthalmia (or, as he terms it, arthritic iritis) is in reality a gouty inflammation—yet he adds, " it is known by many remarkable characters, and is *unquestionably connected* with a peculiar state of the constitution." M. Velpeau maintains that its characters are not peculiar, but merely those of iritis, together with some others, which are referred by authors to inflammation of the choroid membrane. And, speaking of the increased size and varicose state of the vessels, states, that this is merely a consequence of the inflammation—true, but if a mere result of ordinary inflammation, these and other symptoms would take place upon all occasions, which is not the fact. Finally, is it not strange that, while M. Velpeau strenuously maintains that neither scrofula, gout, or rheumatism impress with their own peculiar characters the inflammation developed in the eye, he at once, and without the slightest demur, admits a syphilitic iritis—blepharitis, &c. and proceeds *con gusto* to describe its specific characters and the specific treatment he judges necessary for its cure?

Our Lecturer considers it particularly " absurd to assert that a patient is rheumatic or scrophulous, merely because the ophthalmia by which he is attacked presents certain characters, although he himself may offer no symptom whatever of either the one or the other of these affections." We confess we see no absurdity in this, more than when a knee-joint is attacked with symptoms which have been determined to be rheumatism—or a young boy's glands swell and suppurate a cheesy matter—asserting that fact, and naming the disease rheumatic or scrofulous, although " he himself may offer no symptom whatever of either the one or the other of these affections "—we presume M. Velpeau means no *other* symptom—we are quite content with the full manifestation of a disease in all its stages in one part or *organ!* and certainly do not feel warranted in denying the existence of a disease because it is not developed *cap-a-pie*, furnishing to the senses every manifestation in every part ever noted or described as evidencing " rheumatism " —" scrofula "—or " gout." For we observe M. Velpeau's objection is not that certain characters are wanting—but that the particular diseases in question content themselves with their development in one organ! Thus we find his objection removed as soon as he finds these affections elsewhere—he is quite willing " to allow that rheumatism or scrofula, co-existing with an inflammatory affection of the eye, exercises more or less influence over that affection "—that is, he is willing then to admit what he had so lately termed " absurd,"—viz. a rheumatic affection of the eye!

But the arguments throughout the lecture on the subject are but loosely stated, and present many very obvious contradictions—nor should we have gone at all into their analysis but for the dangerous principle enforced, that such distinctions have no practical utility, and the treatment should have no reference to them.

Then he teaches that these affections of the eye should be treated locally and seldom requires general measures—we will venture to affirm, without fear of contradiction, that if he does so, three-fourths of such patients are never cured by his treatment—that no local remedies hitherto used will remove these specific affections. But a little further on, what is our surprize to find this system entirely abandoned, and M. Velpeau declaring that, " When there is any constitutional predisposition, *such as scrofula, I always have recourse to those therapeutic agents which are calculated to modify it!*"

The long flourish of originality of opinions—maintaining the non-existence of these specific affections of the eye, and the futility of a treatment based upon the opinion that they exist, only serves to usher in—after innumerable contradictory statements, well calculated to confuse the uninitiated—a treatment precisely analogous to that adopted by surgeons who admit the existence of inflammatory affections of peculiar and specific characters.

After all this, our Lecturer, unembarrassed by his previous admissions and denials, commences a new section, and, with much sang-froid, tells us, " that an attentive and careful examination of the inflammatory diseases by which the eye is attacked, has shown us that those forms of ophthalmia which authors have denominated *catarrhal, arthritic, rheumatic*, and *scrophulous*, do not present any thing specific either in their symptoms or in their treatment, (!) but this is no longer true with regard to syphilitic ophthalmia; " for it is an undeniable fact, that syphilis does impress on inflammatory affections of the eye, peculiar characters which require a specific treatment." Again, " we cannot but admit that it is perfectly rational to admit the existence of syphilitic ophthalmia." Yet M. Velpeau has said it is perfectly absurd to admit the existence of specific diseases of the eye—that they have no practical utility, and are mischievous, inasmuch as they prevent the profession treating them as simple inflammations of the various tissues.

We must leave it to the Lecturer, then, to reconcile his own opinions and practice, with a free confession of our inability to accomplish the task for him.

On the subject of iritis, where there is much better evidence of judgment and careful observation, we do not remark much difference between the doctrines inculcated and those generally developed in the English schools.

The credit of the first accurate description of iritis, and consequent improvement in the treatment, is given with candour to the German and English surgeons—Beer, Schmidt, and nearly about the same time Ware and Saunders.

A distinction is made—not original, certainly, but still scarcely sufficiently dwelt upon in English practice, we are inclined to believe, between primitive and secondary iritis—" between the inflammatory affection which commences by the iris, and that which is merely a consequence of the inflammation of some other organ."

"This distinction is important, as secondary iritis will often disappear when the affection by which it is caused has given way, and is often much less difficult to cure than primitive iritis. Local remedies also sometimes prove successful in secondary iritis, whereas they have little or no influence over the primitive form of inflammation."

With respect to the administration of mercury as a purgative, an alterative, and a sialogogue, M. Velpeau has neither, like some of his countrymen, gravely adopted the opinions of English surgeons; nor, like others, does he seem prepared to disclaim them. Judging from the opinion he gives, we must believe that his experience of the action of this remedy as a cause of mercurial action in the system, has been by no means great.

"I have very frequently given calomel, both as a purgative, as a mercurial agent, and as an alterative; and I must say, that I have sometimes seen the iritis disappear when the economy was deeply disturbed by its action. True, I have seen the decrease and final disappearance of the inflammation coincide with salivation, but I am not prepared to say whether this was the result of the medication I was employing—or whether it was mere coincidence."

We think, if M. Velpeau will give it a more extended trial, he soon will be prepared to say, with the majority of English surgeons, that mercurial action is perfectly capable of arresting the inflammatory action in the iris—that it does so in a great proportion of cases—although such a result does not invariably take place. And of all the remedial agents employed for the cure of iritis, it is the one upon which surgeons may most frequently rely. M. Velpeau has tried the turpentine but rarely—doubts its efficacy theoretically, and seems little disposed to resort to its exhibition.

Upon the whole, these lectures prove that there is little information we possess on the subject of which they treat, not possessed by our neighbours, little indeed that is not mutually shared. That, with the exception of some variations, such as we have brought before our readers, as to treatment, and a few peculiar opinions which do not always seem to us to be founded upon any well-sustained or deep conviction, there is no great or striking difference in the ophthalmic surgery of the two countries.

The chief points of difference as to the *nature* of the diseases, and which we have noticed at some length, are the existence of a granular conjunctival inflammation apart from the purulent or catarrhal—the different nature of the purulent ophthalmia of infants, and the Egyptian—the non-existence of arthritic, scrophulous or rheumatic ophthalmia—admitting as the sole specific disease of the eye the syphilitic.

These are, we find, the principal features, separating the principles and practice inculcated in these lectures, from those taught in our own schools.

As these lectures do not include the operations, we shall not at present extend our comparison beyond those diseases to which we have already directed attention.

